# Smad3 deficiency promotes beta cell proliferation and function in *db/db* mice *via* restoring Pax6 expression

**DOI:** 10.7150/thno.51857

**Published:** 2021-01-01

**Authors:** Jingyi Sheng, Li Wang, Patrick Ming-Kuen Tang, Hong-Lian Wang, Jian-Chun Li, Bi-Hua Xu, Vivian Weiwen Xue, Rui-Zhi Tan, Nana Jin, Ting-Fung Chan, Xiao-Ru Huang, Ronald CW Ma, Hui-Yao Lan

**Affiliations:** 1 Department of Medicine and Therapeutics, Li Ka Shing Institute of Health Sciences, The Chinese University of Hong Kong; 2 Research Center for Integrated Chinese and Western Medicine, and Department of Cardiology, The Second Affiliated Hospital, Southwest Medical University, Luzhou, Sichuan, China; 3 Department of Anatomical and Cellular Pathology, State Key Laboratory of Translational Oncology, The Chinese University of Hong Kong; 4 State Key Laboratory of Bioelectronics, Jiangsu Key Laboratory for Biomaterials and Devices, School of Biological Sciences & Medical Engineering, Southeast University, Nanjing, China; 5 School of life science, the Chinese University of Hong Kong, Hong Kong SAR, China; 6 Guangdong-Hong Kong Joint Laboratory on Immunological and Genetic Kidney Disease, Guangdong Academy of Medical Sciences, Guangdong Provincial People's Hospital, Guangzhou, China.

**Keywords:** Type 2 diabetes, Islet beta cells, Smad3, Pax6

## Abstract

**Rationale:** Transforming Growth Factor-beta (TGF-β) /Smad3 signaling has been shown to play important roles in fibrotic and inflammatory diseases, but its role in beta cell function and type 2 diabetes is unknown.

**Methods:** The role of Smad3 in beta cell function under type 2 diabetes condition was investigated by genetically deleting Smad3 from *db/db* mice. Phenotypic changes of pancreatic islets and beta cell function were compared between Smad3 knockout *db/db* (Smad3KO-*db/db*) mice and Smad3 wild-type *db/db* (Smad3WT-*db/db*) mice, and other littermate controls. Islet-specific RNA-sequencing was performed to identify Smad3-dependent differentially expressed genes associated with type 2 diabetes. *In vitro* beta cell proliferation assay and insulin secretion assay were carried out to validate the mechanism by which Smad3 regulates beta cell proliferation and function.

**Results:** The results showed that Smad3 deficiency completely protected against diabetes-associated beta cell loss and dysfunction in *db/db* mice. By islet-specific RNA-sequencing, we identified 8160 Smad3-dependent differentially expressed genes associated with type 2 diabetes, where Smad3 deficiency markedly prevented the down-regulation of those genes. Mechanistically, Smad3 deficiency preserved the expression of beta cell development mediator Pax6 in islet, thereby enhancing beta cell proliferation and function in *db/db* mice *in vivo* and in Min6 cells *in vitro*.

**Conclusions:** Taken together, we discovered a pathogenic role of Smad3 in beta cell loss and dysfunction via targeting the protective Pax6. Thus, Smad3 may represent as a novel therapeutic target for type 2 diabetes prevention and treatment.

## Introduction

Type 2 diabetes affects people worldwide with 10-20% mortality 1-3. Its onset and progression are multifactorial but largely due to the development of systemic insulin resistance and insufficient insulin secretion 1-3. Islet beta cells are the sole source of insulin, therefore malfunction of them is a key for type 2 diabetes onset 4. Indeed, deterioration of beta cell development and function is commonly observed in type 2 diabetes patients and experimental diabetic models 3, 5. Interestingly, increasing studies suggested that beta cell development can be recovered at the prediabetic stage 4. Thus, elucidation of the pathogenic mechanism of diabetic islet regression at early stage would discover potential therapeutic targets for type 2 diabetes prevention and treatment.

TGF-β/Smad signaling is a well-known regulator of fibrosis and inflammation, but its role in diabetes has been less elucidated 6-9. Under diabetic condition, the elevated levels of TGF-β, advanced glycation end-products (AGEs) and angiotensin II (Ang II) can activate Smad3 in different tissues and organs 10-14. Our previous work has demonstrated a key role of Smad3 in mediating diabetic kidney injury via transcriptional regulation 15-17. Multiple evidences have suggested a role of TGF-β/Smads signaling in regulating islet development and function. A Smad signaling network had been reported for regulating islet beta cell proliferation in mice receiving partial pancreatectomy (PPx) 18. In addition, Dhawan *et al.* demonstrated that inhibition of TGF-β signaling promotes human pancreatic beta cell replication in nondiabetic mice via suppressing Smad3 19. Furthermore, public data of chrome state suggested a significant association between Smad3 and type 2 diabetes (T2D Knowledge Portal http://www.type2diabetesgenetics.org/), where Smad3 transcriptional activity is highly activated especially in the islet. Nevertheless, the regulatory role and underlying mechanism of Smad3 in diabetic islet development and function are still largely unexplored.

In order to identify the regulatory mechanism of Smad3 in the diabetic islet, we generated Smad3-knockout *db/db* mice with our well-established platform 15, 16. Surprisingly, we found that Smad3 deficiency completely protected against the diabetic-associated islet beta cell loss and insulin insufficiency in *db/db* mice. In addition, we conducted high throughput RNA-seq and revealed the transcriptome profiles of Smad3-WT/KO islets during diabetic development. Eventually, we uncovered a Pax6-dependent protective mechanism of Smad3 deficiency in preserving the proliferation and function of islet beta cells in *db/db* mice. Thus, islet-specific silencing of Smad3 may represent as a novel therapeutic strategy for the type 2 diabetes prevention and treatment.

## Methods

### Animals

Smad3^+/-^ mice on C57BL/6J background were intercrossed with Lepr^+/-^ (db/m) mice on a C57BLKs/J background to produce Smad3^+/-^ db/m heterozygous mice. Double-heterozygous Smad3^+/-^ db/m male and female were intercrossed to generate double mutation of Smad3 and Lepr (Smad3 KO-*db/db*) and control littermates (Smad3 WT-db/m; Smad3 KO-db/m; Smad3 WT-*db/db*; Smad3^+/-^ *db/db*). In Smad3 KO mice, targeted deletion of exon 8 in *Smad3* gene has been shown to delete a portion of the Smad3 C-terminal end which is essential for interacting with TGF-β receptors, resulting in disrupted Smad3 phosphorylation and nuclear translocation 20. All animal husbandry and animal experiments were approved by the Animal Ethics Experimental Committee of the Chinese University of Hong Kong and confirmed to be in accordance with local regulations.

### Fasting blood glucose, body weight, food intake and weight of white adipose tissue (WAT)

Fasting blood glucose levels were measured by Accu-Chek glucose meter (Roche Diagnostics, Indianapolis, IN) in all mice after fasting for 6 h as recommended by the Animal Models of Diabetic Complications Consortium. For the body weight measurement, all mice were weighed individually at indicated time points. To examine the possible influence of daily food-intake on the body weight gain, mice were housed individually, and the amount of daily food-intake was weighed and recorded at the same time of body weight measurement in every 4 or 6 weeks over the 32 week-period. The amount of food-intake (g) against the body weight (g) was then calculated and compared in all mice with different genotypes. In addition, inguinal (I) and epididymal (E) white adipose tissue (WAT) were dissected and weighed in individual mouse when sacrificed at 20 weeks of age.

### Glucose and Insulin Tolerance Tests

For glucose tolerance tests, mice were fasted for 6 hours and given i.p. injection of glucose (2 mg/g body weight). Blood glucose levels were determined at 0, 5, 15, 30, 60 and 120 min post injection. For insulin tolerance tests, mice were fasted for 6 hours and given i.p. injection of insulin (1 U/kg). Blood glucose levels were determined at 0, 15, 30, 60 and 120 min post injection.

### H&E staining and quantitation of islet number and area

Pancreas was fixed in 4% paraformaldehyde overnight followed by dehydration in gradual ethanol solution and embedding in paraffin wax. The embedded pancreas was sectioned at 3 μm. H&E staining was performed as previously described. For quantitation of islet number. total islet numbers from five non-consecutive sections were counted for each mouse pancreas and divided by corresponding area. Islet number from at least 5 mice's pancreas for each group were included for analysis. For quantitation of islet area, area of all islets was quantified from five non-consecutive sections of each mouse's pancreas. 5 mice's pancreas were included for each group. All quantitation was performed with Image J software.

### Fluorescent immunostaining

For fluorescent immunostaining, paraffin pancreas section was de-waxed and rehydrated to distilled water. Antigen retrieval was performed by boiling in microwave for 10 min in 0.01 M citrate buffer (pH 6.0). After cooling, the section was blocked in 10% goat serum plus 1% BSA for 1 h at room temperature. Primary antibody incubation was performed at 4˚C overnight. Corresponding secondary fluorescent antibodies were applied to the section for 1 h at room temperature. Cell nuclei were counterstained with DAPI (Sigma, D9542). Image was taken with a fluorescent microscope (ZISS). For staining against Pax6, a 5 min treatment with cold (-20˚C) methanol was included after antigen retrieval. Primary antibodies used in this study were FITC-conjugated mouse anti-insulin (ebioscience, 53-9769-82, 1:200), rabbit anti-glucagon (Abcam, ab93527, 1:1000), rabbit anti Pax6 (Biolegend, 90130 1:300). Secondary antibodies used in this study was Alexa Fluor 555-conjugated goat anti-rabbit antibody (Invitrogen, A27039, 1:400).

### *In vivo* BrdU assay

Mice were i.p. injected with 50 mg/kg/day BrdU solution (dissolved in saline, Sigma, B5002) for 7 consecutive days. Mice were sacrificed 2 h after the last BrdU injection and pancreas were harvested, fixed, embedded and sectioned as above described. BudU immunostaining procedure was the same as above fluorescent immunostaining except that a 30 min treatment of HCl (2 M) was included after antigen retrieval and before blocking. Primary antibodies used were mouse anti BrdU (Dako, M0744, 1:200) and rabbit anti insulin (Abcam, ab181547, 1:100). Secondary antibodies used were Rhodamine-conjugated goat anti-mouse antibody (Chemicon, AP124R, 1:400) and FITC-conjugated goat anti-rabbit antibody (Zymed, 81-6111, 1:400). Cell nucleus were counterstained with DAPI (Sigma, D9542). The proliferation rate in single islet was calculated as percentage of BrdU and insulin double positive cells in insulin positive cell pool. At least three mice were included for each group.

### Isolation and Analysis of Mouse Pancreatic Islets

Islets were isolated from mice as previously described 21, 22. 3 ml of Collagenase V (0.5 mg/ml in HBSS, Sigma, C9263) was infused into the pancreas through the common bile duct as previously described. The infused pancreas was removed and incubated in 2 ml of the same Collagenase V solution at 37 °C for 15 min in water bath followed by shaking to breakdown the tissue and release the islets. The digestion was stopped by adding 20 ml of 1 mM CaCl_2_ (in HBSS) and centrifuged at 290 g for 1 min. The pellet was re-suspended in 5 ml of fresh cold HBSS and passed through a 70-um cell strainer followed by thorough washing with HBSS. Islets retained on the strainer were rinsed into a 10-cm petri dish and picked up with a pasteur pipette under microscope.

### Quantification of beta cell mass

To analyse the β cell mass, three non-consecutive paraffin sections of pancreas from each mouse were stained against insulin and counterstained with eosin. The percentage of β cell area to intact pancreas section area was quantified with Image J software. β cell mass was calculated by multiplying the percentage of β cell area by total pancreatic weight and further normalized by bodyweight.

### RNA-Sequencing and processing

Total RNA was extraction from the isolated islets using miReasy Mini Kit (QIAGEN, 154050997). RNA samples from 3-5 mice per group were pooled into one sample for RNA-sequencing using the Illumina TruSeq Stranded Total RNA Sample Prep Guide library protocol and HiSeq 2500 (Illumina, San Diego, CA, USA) by Macrogen Inc. (Seoul, Korea). FastQC (v0.11.5) was used to ensure the reliability of the 51~95 million 101 bp paired-end raw sequencing reads per sample. Reads were mapped to the GRCm38 assembly using Bowtie 2 (v2.2.6) 23 after trimming the adapters and low quality bases (Q<30 in 4bp sliding window) using Trimmomatic (v0.36) 24. Fragments Per Kilobase of transcript per Million mapped reads (FPKM) for each gene was quantified using RSEM (v1.2.25) 25 with gene annotations in Gencode release M14. Significantly enriched Kyoto Encyclopedia of Genes and Genomes (KEGG) pathways performed using DAVID 26 were defined as FDR < 0.1. DEGs, KEGG and GO analysis data from the RNA-seq were provided in the supplementary files (File name: RNA-Seq DEG Log2FoldChange_by_group, RNA-Seq KEGG_GO_S3KOdbdb *vs.* S3WT dbdb). RNA-seq raw data was deposited to Sequence Read Archive (SRA), NCBI. BioProject ID: PRJNA669969 (http://www.ncbi.nlm.nih.gov/bioproject/669969). The data will be released on 31 Dec 2021 (or upon publication, whichever is first).

### Chromatin immunoprecipitation-Sequencing (ChIP-seq)

To perform ChIP-Seq, a collection of more than 4000 islets was subjected to ChIP procedure with Smad3 antibody (Abcam, ab28379, USA). ChIP was performed with the SimpleChIP® Plus Enzymatic Chromatin IP Kit (CST, 9005, USA) following the recommended procedure. The DNA derived from the ChIP and corresponding input was subjected to high throughput sequencing (Ribo Bio-Tec. China). The library was constructed with the NEBNext® Ultra™DNA Library Prep Kit (NEB, USA) followed by sequencing with the NovaSeq 6000 sequencing system (Illumina, USA). The resulted raw sequencing data were treated with adapter removal and trimming by Trimmomatic software (v0.36). Reads of low quality were detected by FastQC software and excluded for analysis. The resulted clean reads were aligned to mouse genome with Bowtie2 software. Peaks calling were performed by MACS2 software with the corresponding input sample serving as background. The identified peaks were annotated with Homer software. The peak distribution in specified genomic region was visualized in UCSC Genome Browser.

### Real-time PCR

Total RNA was extraction from isolated islets using miReasy Mini Kit (QIAGEN, 154050997) and reverse transcribed using M-MLV reverse transcriptase (Promega). Real-time PCR was performed using SYBR Green (Biorad). Primer sequences are presented in Table S2. Relative gene expression levels were normalized to that of β-Actin.

### Glucose induced Insulin secretion

Min6 cells were seeded in 96 well plate overnight and infected with indicated lentivirus at MOI of 100 for 48 h. Cells were then incubated with Krebs-Ringer bicarbonate HEPES (KRBH) buffer containing 0.2% FBS and 2.8 mM glucose for 2 h, and then were changed to fresh KRBH buffer supplemented with 0.2% FBS, and 2.8 or 25 mM glucose for 30 min. For another experiment, Min6 cells were pre-treated with or without SIS3 (2.5 μg/mL) for 24 h, and stimulated with AGE (100 μg/mL) or TGF-β (1 ng/mL) for another 48 h and cultured with 2.8 mM and 25 mM glucose as described above. Insulin concentrations in the culture supernatant were measured by Insulin ELISA Kit (Crystal Chem, 90080) following manufacture's protocol. Relative insulin secretion in each group was quantified by normalizing the concentration to 2.8 mM glucose-scramble group.

### Min6 cell proliferation assay

Min6 cells were seeded in chamber slide overnight and infected with indicated lentivirus at MOI of 100 for 48 h. Infected cells were treated with advanced glycation end-product (AGE) (100 μg/mL) for another 48 h and cultured with BrdU labeling solution (10 μM) for 4 h. For another experiment, Min6 cells were pre-treated with or without SIS3 (2.5 μg/mL) for 24 h, and stimulated with AGE (100 μg/mL) or TGF-β (1 ng/mL) for another 48 h and cultured with BedU as described above. Cells were fixed and stained as described above for *in vivo* BrdU assay. The proliferation rate in Min6 cells was calculated as percentage of BrdU positive cell numbers of total nucleus numbers in each view field. More than 20 view fields were quantified of each group.

### Bioinformatic analysis using online tools

Public data of Assay for Transposase-Accessible Chromatin using sequencing (ATAC-Seq) from Varshney *et al.* was used to illustrate Smad3 chromatin states and to map the transcription factor biding sites using ChromHMM method in different tissues (adipose tissue, pancreatic islets, liver and skeletal muscle) at T2D Knowledge Portal (http://www.type2diabetesgenetics.org/) 27, 28. The enrichment of active transcription start sites at the Smad3 genomic sequence in islets of type 2 diabetes patients was validated with another online platform of Lawlor *et al.* (https://shinyapps.jax.org/endoc-islet-multi-omics/). The DIAMANTE (European) T2D GWAS was from publicly available human genetic datasets on T2D Knowledge Portal (http://www.type2diabetesgenetics.org/) and was to indicate SNPs overlapping with ATAC-seq footprints.

### Statistical Analysis

Statistical analysis of the differences in fasting serum insulin were performed by two-way analysis of variance (ANOVA), followed by a Newman-Keuls multiple comparisons test using Prism 6.0 (GraphPad Software, San Diego, CA). All other statistical analysis between two groups were performed by one-way analysis of variance (ANOVA), followed by a Newman-Keuls multiple comparisons test using Prism 6.0 (GraphPad Software, San Diego, CA). A *p*-value lower than 0.05 was considered as significant.

## Results

### Smad3 deficiency prevents beta cell loss and insulin insufficiency in type 2 diabetes

**Figure 1:**
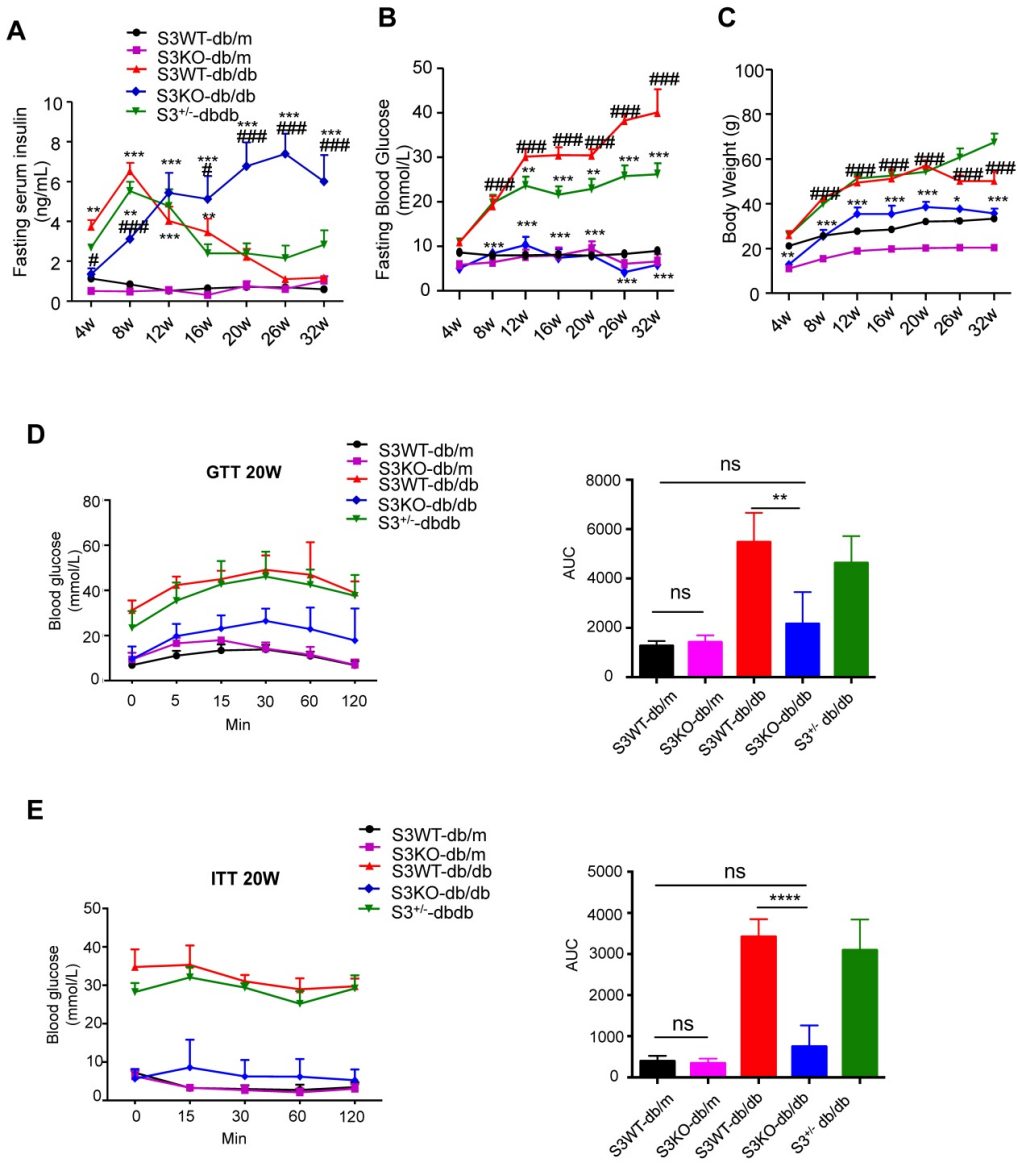
** Smad3 deficiency promotes insulin secretion and protects against type 2 diabetes development in *db/db* mice.** (A-C) Fasting serum insulin (A), Fasting blood glucose (B), and body weight (C) of five groups of mice (n ≥ 8 per group) from 4-32 weeks of age. Data are mean ± S.E.M. ^*^p < 0.05, ^**^p < 0.01, ^***^p < 0.001 *vs.* S3WT-db/m; #*p <* 0.05, ###*p <* 0.001 *vs.* S3WT-*db/db*; two-way ANOVA following Newman-Keuls multiple comparisons. (D-E) Blood glucose levels and area under curve (AUC) during intraperitoneal glucose tolerance test (IPGTT) (D) and intraperitoneal insulin tolerance test (IPITT) (E) at 20 weeks of age (n=5 per group). Data are mean ± S.E.M. ns, no significance, **p < 0.01, ****p < 0.0001 compared between groups as indicated. Statistical analysis was performed by one-way ANOVA, followed by multiple comparisons.

The role of Smad3 in islet beta cell under type 2 diabetes condition was investigated by knocking out Smad3 in the *db/db* mice, which was described in our previous works 15-17. Surprisingly, we found that deletion of Smad3 resulted in a dramatic increase in blood insulin levels over 8-32 weeks of age in Smad3 KO-*db/db* mice (Figure 1A). In contrast, Smad3 WT-*db/db* and Smad3^+/-^ *db/db* mice exhibited a rapid elevation of blood insulin levels from 4 to 8 weeks of age, followed by a steady decline over weeks 8-32 (Figure 1A). In addition, we found that deletion of Smad3 in *db/db* mice protected against the development of hyperglycaemia (Figure 1B), obesity (Figure 1C, Figure S1A-C), glucose intolerance (Figure 1D) and insulin resistance (Figure 1E), while the normalised food intake was not significantly altered in Smad3 KO-*db/db* mice (Figure S1D).

**Figure 2:**
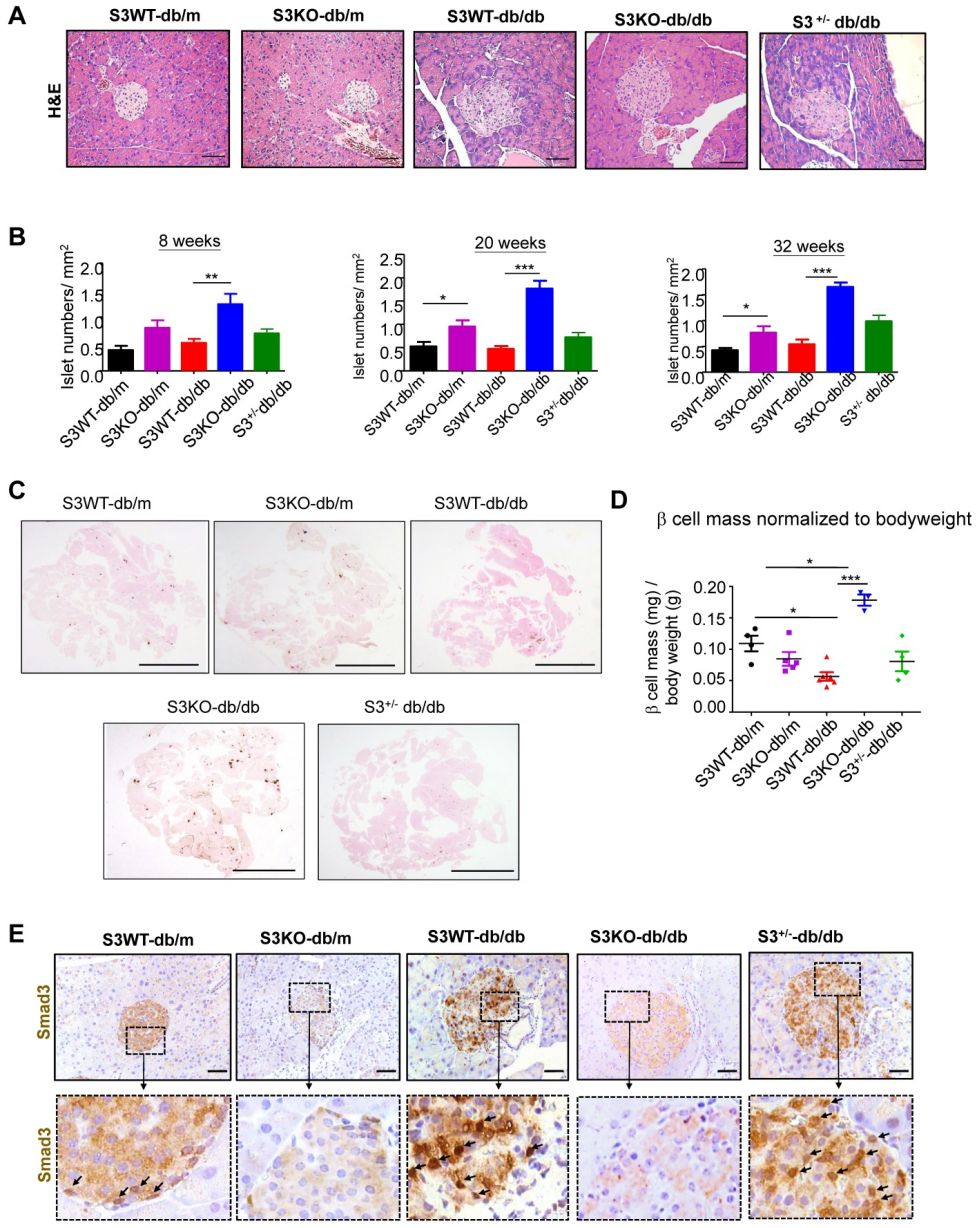
** Smad3 deficiency protect against beta cell loss in *db/db* mice.** (A) Representative images of H&E staining showing pancreas islet morphology differences at 20 weeks of age. Scale bar, 50 μm. (B) Average islet numbers per mm^2^ pancreas area at 8, 20 and 32 weeks of age (More than 6 whole pancreas sections were quantified per group). (C) Representative images of immunohistochemistry (IHC) staining against insulin in the pancreatic section to show the islet β cells. Scale bar, 5 mm. (D) Quantification of β cell mass in pancreas normalized to bodyweight. Each dot represents one animal. Data are mean ± S.E.M. *p < 0.05, **p < 0.01, ***p < 0.001 compared between groups as indicated. Statistical analysis was performed by one-way ANOVA, followed by multiple comparisons. (E) Representative images of IHC staining showing Smad3 hyperactivation (nuclear translocation) in S3WT-*db/db* islet. Scale bar, 50 μm.

The huge differences in mouse serum insulin levels suggest that deletion of Smad3 may have an impact on islet beta cells in type 2 diabetes. As shown in Figure 2A and Figure S2, Smad3 KO-*db/db* mice appeared to have enlarged islet sizes with normal islet morphology and architecture, while islets in Smad3 WT-*db/db* and Smad3^+/-^ *db/db* mice exhibited detectable pathological changes including islet cells swelling, degeneration, and acinar cells infiltration (Figure 2A, Figure S2). In addition, *db/db* mice lacking Smad3 exhibited markedly increased numbers of pancreatic islets (Figure 2B), the volume of beta cell mass (Figure 2C-D), and the average cell numbers per islet (Figure S3) as compared to Smad3 WT-*db/db* mice. Importantly, immunohistochemistry revealed that these pathological changes in the islets of Smad3 WT-*db/db* and Smad3^+/-^ *db/db* mice were apparently associated with increased Smad3 nuclear translocalization (Figure 2E), implying that Smad3 signaling was highly activated in the islets under diabetic condition. These results suggested that hyper-activated Smad3 signaling may be one of the causes for the beta cell phenotypic change in *db/db* mice.

**Figure 3:**
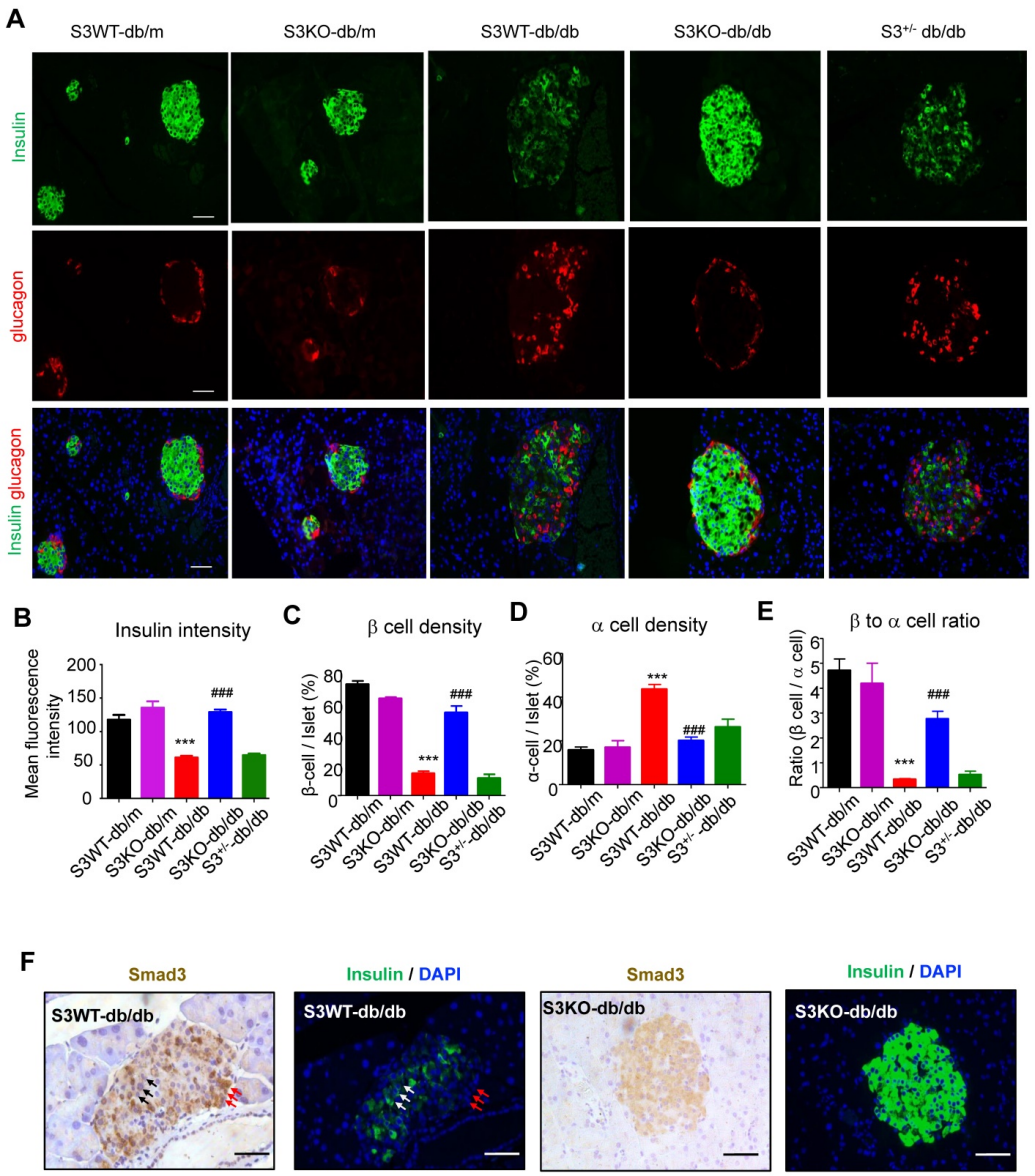
** Smad3 deficiency restores insulin production in beta cells of *db/db* mice.** (A) Representative images of immunofluorescence (IF) staining of beta cells (insulin-positive; green), and alpha cells (glucagon-positive; red) in pancreas islets at 20 weeks of age. Nuclei were stained with DAPI (blue). Scale bar, 50 μm. (B) Quantification of insulin intensity in beta cells. (C-E) Beta (C) and alpha (D) cells were quantified as a percentage of total islet area. Beta to alpha cell ratio (E) was quantified as the ratio of beta area to alpha cell area (More than 20 islets were quantified per group). ***p < 0.001, *vs.* S3WT-db/m; ###* p <* 0.001, *vs.* S3WT-*db/db*. (F) Consecutive sections with IHC and IF staining showing Smad3 hyperactivation (nuclear translocation) negatively associated with insulin expression (green) in S3WT-*db/db* islet. Scale bar, 100 μm.

We next investigated the regulatory role of Smad3 in islet beta cells function. Immunofluorescence detected that in Smad3 WT-*db/db* and Smad3^+/-^ *db/db* mice, mean insulin intensity was largely decreased as compared to non-diabetic controls, while deletion of Smad3 completely restored insulin staining intensity in *db/db* mice (Figure 3A-B). In addition, recovered beta cell percentage, alpha cell percentage and beta to alpha cell ratio were found by Smad3 deficiency in *db/db* mice (Figure 3C-E). Moreover, consecutive section staining of Smad3 and insulin showed that Smad3 activation (nuclear localisation) was negatively associated with insulin expression in the islet cells (Figure 3F), implying a suppressive role for Smad3 in islet beta cell function. Collectively, these results support the notion that Smad3 deficiency largely increased beta cell mass and promoted beta cell function in *db/db* mice.

### Smad3 suppresses beta cell signature genes in type 2 diabetes islet

**Figure 4:**
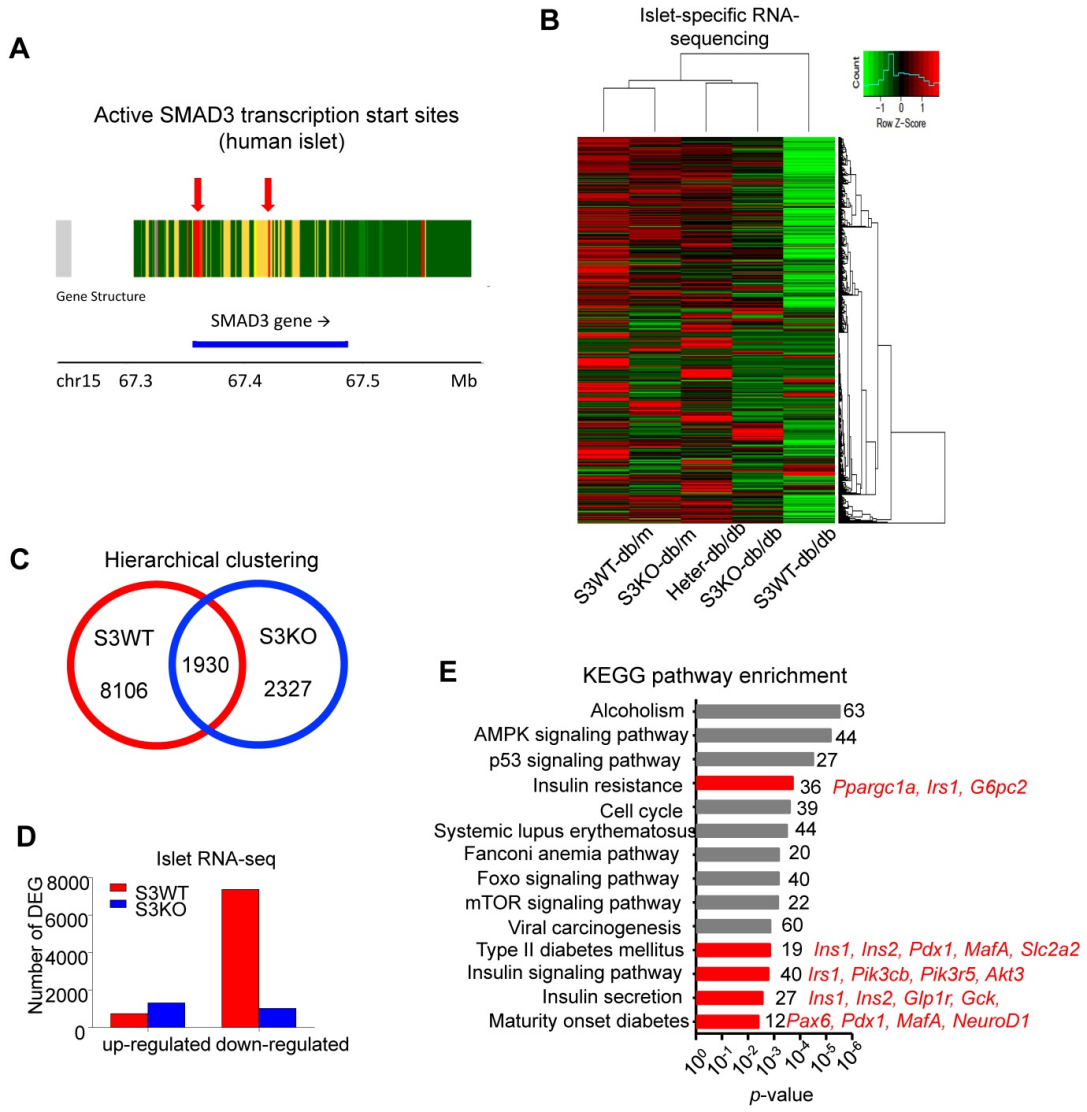
** Smad3 deficiency upregulates beta cell signature genes in diabetic islets.** (A) Enrichment of active transcription start sites at the Smad3 genomic sequence in islet of type 2 diabetes patients analysed with online platform of Lawlor *et al*. (https://shinyapps.jax.org/endoc-islet-multi-omics/). (B-D) High throughput RNA sequencing elucidated the underlying mechanism of Smad3 in diabetic islet development. A distant transcriptome profile of Smad3-WT *db/db* islets compared to other 4 groups showing by (B) heatmap, where (C) Smad3-WT and Smad3-KO dependent DEGs were identified with (D) a markedly down-regulation of DEGs in Smad3-WT group was observed. (E) KEGG analysis showing Smad3-KO dependent up-regulated DEGs were significantly enriched in type 2 diabetes and insulin related pathways (red highlighted) by gene ontology analysis.

In order to examine whether there is any human evidence of a link between Smad3 and type 2 diabetes islet, we explored chromatin accessibility at T2D Knowledge Portal based on public data of ATAC-Seq from Varshney *et al.* in different tissues (Figure S4). Interestingly, we found the abundancy of active transcription start site and enhancer on Smad3 gene was especially increased in pancreatic islets of type 2 diabetes patients as compared to their liver, adipose tissue, and skeletal muscle according to the chromatin states data (Figure S4). The result was also confirmed with another online platform of Lawlor *et al.* (https://shinyapps.jax.org/endoc-islet-multi-omics/) (Figure 4A), suggesting the enrichment of active transcription start sites at the Smad3 genomic sequence in islet of type 2 diabetes patients is valid across different databases. These results suggest an increased potential of transcription factor (TF) binding events and higher transcriptional regulatory potential of Smad3 in type 2 diabetes pancreatic islets.

We next elucidated the regulatory role of Smad3 in islets by conducting islet-specific RNA-sequencing with samples isolated from five genotypes of mice (Smad3-WT/KO db/m and Smad3-WT/KO/Heter *db/db*). Interestingly, the transcriptome profile of Smad3-WT *db/db* mice was significantly distinguished from other groups (Figure 4B). There were 8106 Smad3-dependent differentially expressed genes associated with type 2 diabetes, where Smad3-KO largely decreased the number of down-regulated genes in *db/db* mice (Figure 4C-D). Importantly, KEGG analysis revealed that the upregulated genes in Smad3 KO-*db/db* islets were significantly enriched in the clusters of 'Insulin resistance' (*Ppargc1a*, *Irs1*, and *G6pc2*), 'Type II diabetes mellitus' (*Ins1*, *Ins2*, *Pdx1*, *MafA*, and *Slc2a2*), 'Insulin signaling pathway' (*Irs1*, *Pik3cb*, *Pik3r5*, and *Akt3*), 'Insulin secretion' (*Ins1*, *Ins2*, *Glp1r*, *Gck*, and *Cacna1c*), and 'Maturity onset diabetes of young' (*Pax6*, *Pdx1*, *MafA*, and *NeuoD1*); implying an inhibitory role of Smad3 in multiple beta cell signature genes at transcriptional level (Figure 4E).

### Smad3 directly binds to Pax6 and suppresses its expression in mouse islets

**Figure 5:**
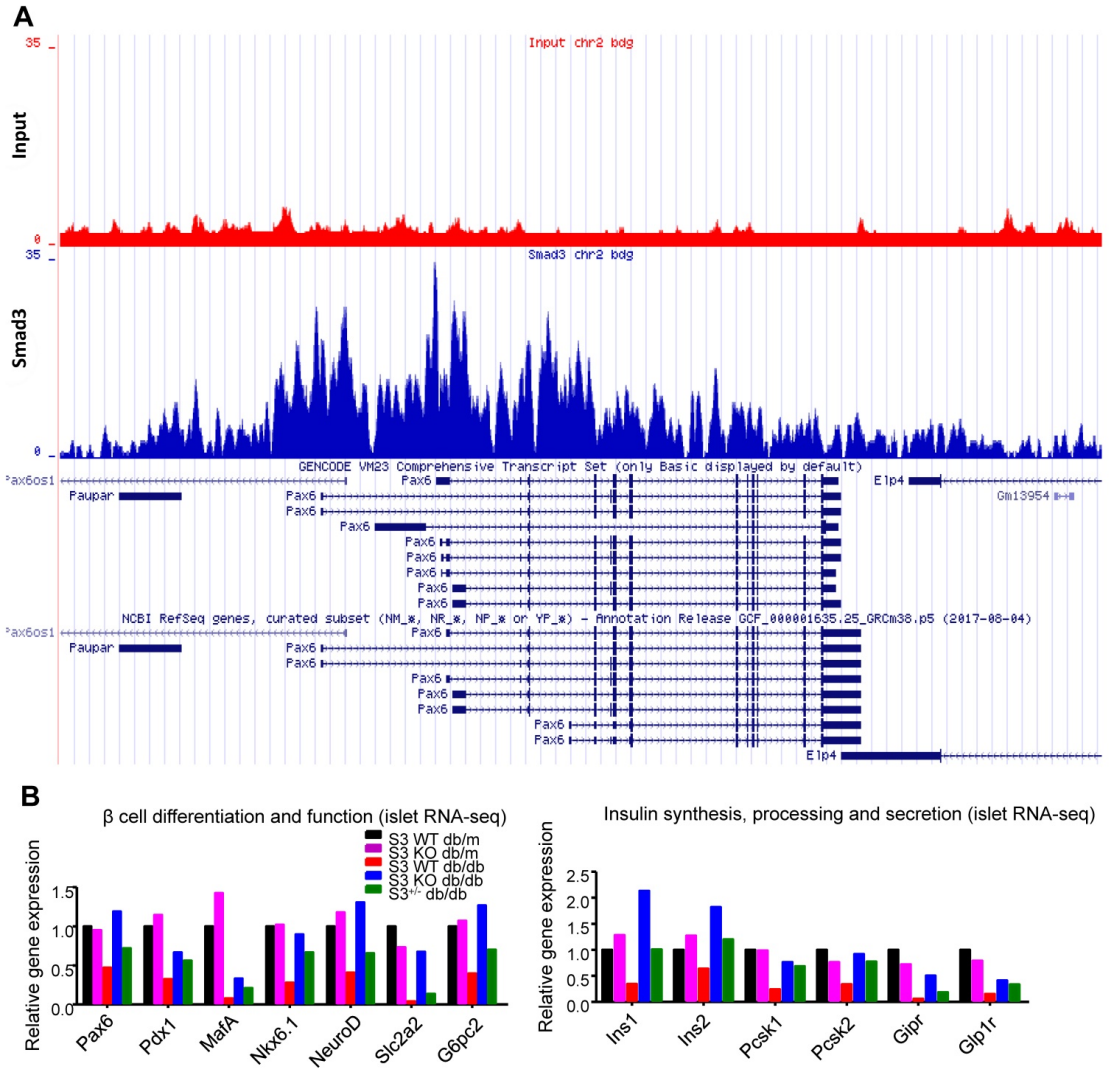
** Smad3 regulates Pax6 and its target genes expression in the islets of *db/db* mice.** (A) ChIP-seq revealing enriched Smad3 binding signal in the regulatory region of Pax-6 genomic locus in mouse islets. (B) RNA-seq revealing the up-regulation of Pax6 and its targeting genes (*Pdx1*, *MafA*, *Nkx6.1*, *NeuroD*, *Slc2a2*, *Ins1*, *Ins2*, *Pcsk1*, *Pcsk2*, *Gipr*, and *Glp1r*) expression by Samd3 deficiency in the islets of *db/db* mice.

**Figure 6:**
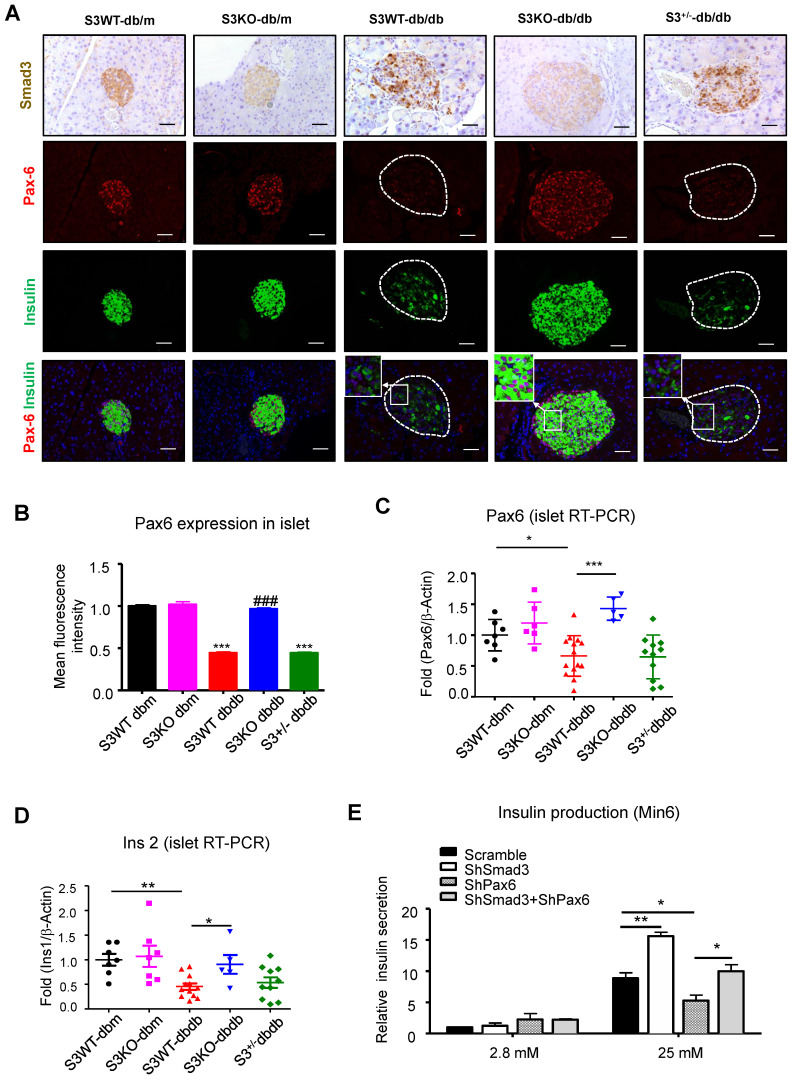
** Smad3 deficiency restores beta cell function via a Pax6-depedendent mechanism.** (A) Representative images of immunofluorescence staining showing a marked reduction in Pax6 expression (red) in the islets of 20-week-old *db/db* mice associated with the decreased insulin production (green), which was blocked by Smad3-KO. (B-D) Quantification of Pax6 staining in islets (B) and confirmed by real-time PCR (C) (n ≥ 5 per group). (D) Real-time PCR confirmed that Smad3 deficiency restored the expression of ins2 in *db/db* mice (n ≥ 5 per group). (E) Knockdown of Smad3 effectively enhanced glucose stimulated insulin secretion in Min6 cells via a Pax6-dependent manner, detected by ELISA *in vitro* (n=4). Data are mean ± S.E.M. (B) ***p < 0.001, *vs.* S3WT-db/m, ### *p <* 0.001, *vs.* S3WT-*db/db*. (C-E) *p < 0.05, **p < 0.005, ***p < 0.001, compared between groups as indicated. One-way ANOVA following multiple comparisons. Scale bar, 50 μm.

Among all the Smad3-dependently suppressed beta cell mediators (Figure 3F), Pax6 is a transcription factor critical for pancreas development and beta cell identity maintenance (Table S1). To investigate whether Smad3 directly regulates Pax 6 transcription in mouse islets, we performed ChIP-seq with Smad3 antibody in mouse islets. As shown in Figure 5A, significantly enriched Smad3 binding signals were found in the regulatory region of Pax-6 genomic locus, providing direct evidence of the transcriptional regulatory role of Smad3 on Pax-6 in islets. In addition, according to our islet-specific RNA-seq results, the expression levels of a dozen of Pax6 targeting genes (Table S1) were significantly recovered by Smad3 deletion in *db/db* mice (Figure 5B). These Pax6 targeting genes were critical beta cell mediators that regulate 'beta cell differentiation and function' and 'Insulin synthesis, processing and secretion', which were largely suppressed in the Smad3-WT *db/db* mice compared to their non-diabetic controls (Figure 5B). The expression levels of Pax6 and its targeting genes were further validated by real-time PCR in isolated islets (Figure 6C&D, Figure S5). Taken together, our findings demonstrated the regulatory role of Smad3 on Pax6 and its targeting genes at the transcriptional level.

Furthermore, immunofluorescence staining confirmed that Pax6 expression was dramatically inhibited in Smad3-WT and heterozygous *db/db* mice compared to the db/m controls, which was completely prevented by Smad3-KO (Figure 6A-B). Consecutive section staining of Smad3 and Pax6/insulin confirmed that Smad3 hyper-activation was associated with reduced Pax6 and insulin expression in *db/db* mice, while Smad3-KO significantly recovered the expression of Pax6 and insulin in *db/db* mice (Figure 6A).

### Smad3 inhibits beta cell proliferation and function via Pax6-depedendent mechanism

**Figure 7:**
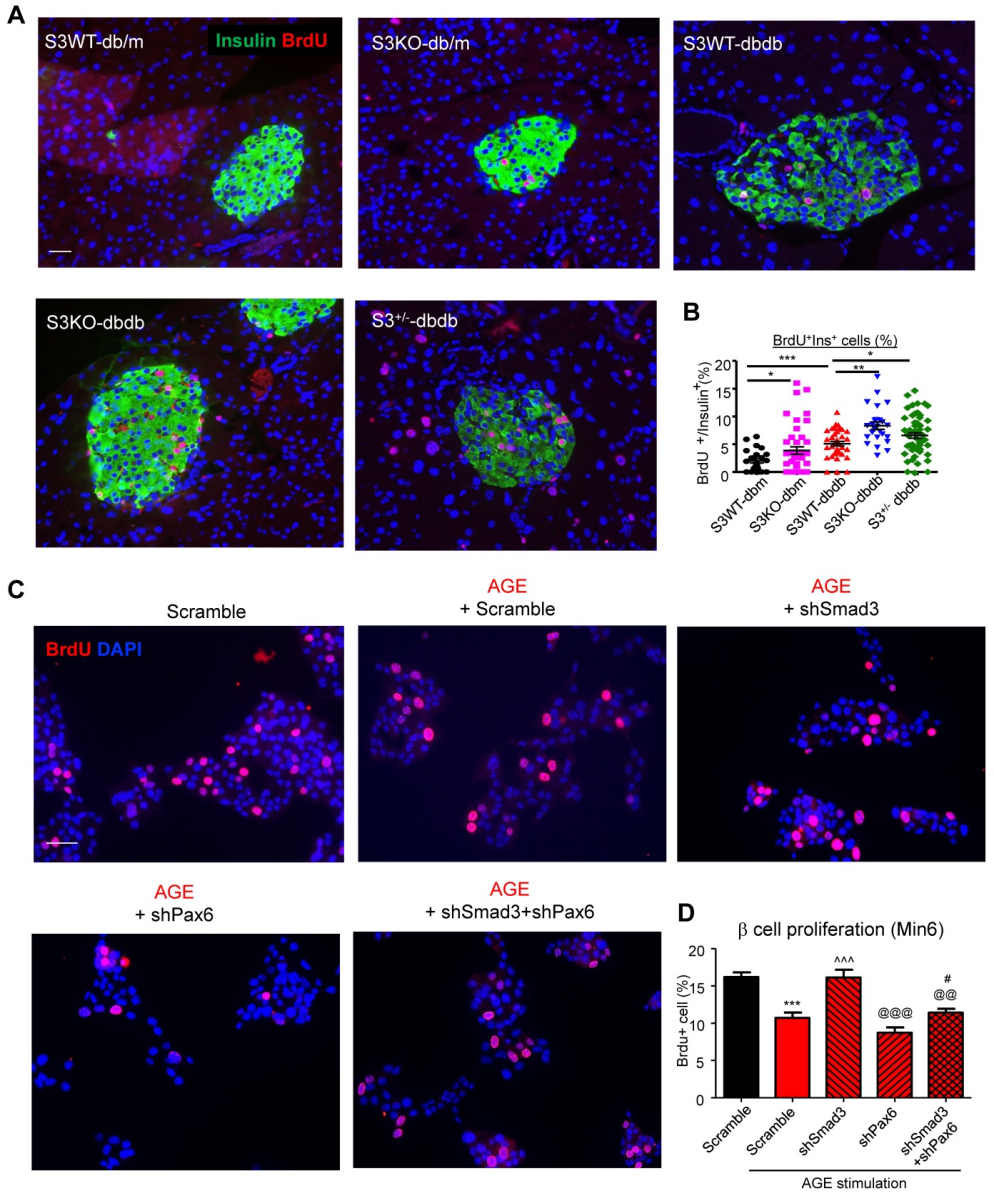
** Smad3 deficiency promotes beta cell proliferation via a Pax6-dependent mechanism.** (A) Representative images of immunofluorescence staining and quantification showing a markedly increased level of BrdU (red) incorporation in the islet cells of 20-week-old Smad3-KO *db/db* mice as compared to Smad3WT-*db/db* mice. (B) Replicating β cells were identified as BrdU^+^Insulin^+^ cells and quantified as percentage of total Insulin^+^ cells (β cells) in the islet. Each point indicates one islet. *p < 0.05, ** *p <* 0.01, **** *p <* 0.0001, compared between groups as indicated. Scale bar, 50 μm. (C-D) Knockdown of Smad3 effectively enhanced Min6 cell proliferation under AGE stimulation in a Pax6-dependent manner, detected by genome-incorporated BrdU level (red) with immunofluorescence *in vitro* (n=4). Scale bar, 50 μm. Data are mean ± S.E.M. ***p < 0.001 *vs.* Scramble; ^^^*p <* 0.001 *vs.* AGE+Scramble; ^@@^*p <* 0.01, ^@@@^*p <* 0.001 *vs.* AGE+shSmad3; #* p <* 0.05 *vs.* AGE+shPax6. One-way ANOVA following multiple comparisons. Scale bar, 50 μm.

Furthermore, we found that Smad3-KO significantly enhanced islet beta cell proliferation in *db/db* mice (Figure 7A). To validate the role of Smad3 in beta cell proliferation, *in vitro* assay was performed by activating Smad3 signaling in Min 6 cells. Our previous publication has reported that AGE is capable of inducing phosphorylation of Smad2/3 in a TGF-β in-dependent manner 13. Here, we found that both AGE and TGF-β impaired cell proliferation in Min6 cells, which was blocked by the pretreatment of SIS3 pretreatment (Figure S6A&B). It was further confirmed that Smad3 deficiency enhanced β cell proliferation in AGE-stimulated Min6 via a Pax-6 dependent manner (Figure 7B, Figure S7).

In addition, both AGE and TGF-β also impaired glucose-stimulated insulin secretion in Min6 cells, which was also inhibited by SIS3, a Smad3 specific inhibitor (Figure S5C). More importantly, *in vitro* assay showed that silencing of Smad3 enhanced high glucose induced insulin production in Min6 cells, while silencing of Pax6 prevented its insulin production, which was further abolished by the dual-silencing with Pax6 (shSmad3+shPax6) (Figure 6E and Figure S7). Taken together, the phenotypic changes in Smad3 KO *db/db* mice and *in vitro* results in Min6 cells suggested a Pax6-dependent mechanism by which Smad3 signaling regulates beta cell proliferation and function.

## Discussion

In this study, we discovered the pathogenic role of Smad3 in type 2 diabetes beta cell loss and dysfunction via the Pax6-dependent mechanism. Our data showed that genetic deletion of Smad3 largely increased the serum insulin levels, and prevented metabolic abnormalities of *db/db* mice, characterized by their normalized blood glucose level, body weight, glucose tolerance, insulin sensitivity, that were comparable to the levels of nondiabetic db/m control mice. Interestingly, we observed an increased regulatory potential of Smad3 in pancreatic islets of type 2 diabetes patients as compared with adipose tissue, liver and skeletal muscle by public ATAC-seq data at T2D Knowledge Portal, while Smad3 hyperactivation was validated in *db/db* mice islets by immunohistochemistry staining. Importantly, we found that deletion of Smad3 completely protected against beta cell loss and dysfunction in *db/db* mice islets. By islet-specific RNA-seq analysis, we revealed that Smad3 deficiency largely promoted the expression of genes that regulates beta cell differentiation and function. By ChIP-seq in mouse islets, we found a direct binding of Smad3 to the *Pax6* gene locus, which is the key beta cell regulating gene. A dozen of Pax6 targeting genes were also found to be significantly downregulated in *db/db* islets and completely recovered by Samd3 deletion. Thus, our findings suggested Smad3 may represent as a novel therapeutic target for type 2 diabetes prevention and treatment.

Previous studies have demonstrated that TGF-β1 has an inhibitory role in pancreas development. Mice overexpressing dominant-negative TGF-β receptor II (DNTβRII) exhibit increased proliferation of pancreatic acinar cells 29, whereas TGF-β1 overexpression in beta cells results in reduced islet size 30. TGF-β1 is able to downregulate a panel of genes related to beta cell function including *Pdx-1*, *Ins1*, *Ins2*, *Nkx6.1*, *Glp1-R* and *NeuroD1* 31, where insulin was identified as a direct target of Smad3 31. In addition, Smad3 transcriptionally suppressed insulin production of islet beta cells *in vitro* 32, which implies that Smad3 might be involved in the islet-mediated type 2 diabetes development at transcriptional level. Therefore, better understanding of the TGF-β/Smad3 signaling in islet development may uncover new therapeutic strategy for type 2 diabetes.

In the current study, we demonstrated that *db/db* mice lacking Smad3 were protected against the development of type-2 diabetic phenotype, characterized by a normal body weight without severe obesity, normal levels of fasting blood glucose, no glucose intolerance nor insulin resistance. The overall phenotype changes in multiple tissues and organs may be regulated by different pathways, although the precise mechanisms are largely unclear. In terms of obesity, it has been shown that Smad3 deficiency can promote mitochondrial biogenesis by increasing proliferator-activated receptor β/δ and decreasing proliferator-activated receptor γ expression in adipocytes 33, 34, resulting in the protection against obesity induced by a high-fat diet. Here we also showed that deletion of Smad3 protected against glucose intolerance and insulin resistance in *db/db* mice. In particular, Smad3 KO-*db/db* mice developed hyperinsulinemia through the lifetime but not insulin resistance, suggesting that Smad3 might also modulates peripheral glucose metabolism and insulin action in a unique manner. Indeed, Smad3 is known to repress the activation of Akt signalling under high TGF-β1 conditions, which is reversed when Smad3 gene is disrupted 35. It is plausible that the interactions of TGF β /Smad3 with PI3K/AKT singaling pathways led to impaired insulin signaling in the periphery, which may partly contribute to the insulin resistance in *db/db* mice.

As reported in previous studies and this study, *db/db* mice exhibited a rapid hyperinsulinemia up to 12 weeks of age, followed by a continuous decrease of blood insulin levels due to a gradual decline of β cell mass, resulting in the development of severe hyperglycaemia 36, 37. When sensing a higher demand for insulin, pancreatic β cells may first undergo a compensation process to produce more insulin, and when β cells fail to sustain this compensatory response, hyperglycaemia and severe T2D develops 38, 39. It is possible that Smad3 KO-*db/db* mice developed a long term β cell compensation in response to a moderate increase in metabolic load. In contrast to Smad3 WT-*db/db* mice, whose inadequate β cell compensation ultimately led to the development of diabetes, the β cell proliferation and function in Smad3 KO-*db/db* mice were well sustained and the compensation was maintained through the lifetime.

Importantly, this paper is the first to report that Smad3 deficiency markedly protected against diabetic islet beta cell loss and dysfunction in *db/db* mice, implying a negatively regulatory role for Smad3 in beta cell proliferation and function. Consistent with previously reported 40, the beta cell failure in type 2 diabetes was not due to the cell death, as less than 1% beta cell with apoptosis was found in islets of both Smad3KO *db/db* and Smad3WT *db/db* (data not provided). On the contrary, Smad3KO *db/db* mice exhibited significantly higher proliferating activities as demonstrated by doubling the islet and beta cell numbers with a 2-3 fold increase in the Brdu^+^Insulin^+^ cells compared to Smad3WT*db/db* mice (Figure 7A), implying that increased beta cell proliferation, but not cell death, may be a mechanism contributing to the beta cell mass changes in Smad3 KO *db/db* mice.

By islet-specific RNA-seq, we revealed the unique Smad3-WT and Smad3-KO transcriptome profiles associated with type 2 diabetes development. We detected a significant upregulation of beta cell signature genes in the islets of Smad3-KO *db/db* mice. Among these genes, Pax6 is found to have multiple Smad3 binding sites, and ChIP-seq directly demonstrated the enrichment of Smad3 binding signal in the regulatory region of *Pax-6* genomic locus in mouse islets. Pax6 is a well-known beta cell regulator that maintains beta cell identity and function by directly or indirectly suppressing multiple genes that are crucial for beta cell differentiation and functions (*Pdx1*, *MafA*, *Nkx6.1*, *Slc2a2*, and *G6pc2*), insulin synthesis (*Ins1* and *Ins2*), proinsulin processing (*Pcsk1* and *Pcsk2*), and glucose-stimulated insulin secretion (*Gipr* and *Glp1r*) 41, 42. Here, we demonstrated the inhibitory effects of Smad3 in beta cell proliferation and insulin production via a Pax6-dependent manner in *db/db* mice *in vivo* and Min6 cells *in vitro*. Thus, transcriptional inhibition of the Pax6 in beta cells may represent as one of the key pathogenic mechanism for the Smad3-driven beta cell loss and dysfunction in type 2 diabetes.

There are some limitations of the study. First of all, the use of global knock out model instead of islet-specific knock out may bring in some confounding factors, such as developmental problems and whole animal pathophysiology, which may cause secondary effects on beta cells. Thus, inducible beta cell-specific Smad3 KO mice with established islet injury in *db/db* mice could be a better approach in future studies to validate these findings. Nevertheless, the Min6 *in vitro* data in this study is supportive to show a direct cause-effect relationship of Smad3/Pax6 axis and beta cell function. In addition, public ATAC-seq data also revealed a more active transcriptional regulatory potential of Smad3 in pancreatic islets of type 2 diabetes patients as compared with adipose tissue, liver and skeletal muscle. Importantly, Smad3KO *db/db* mice exhibited a huge increase in beta cell mass (2-3 times) and serum insulin level (10 times) as compared to non-diabetic controls (Smad3 WT db/m), although their fasting blood glucose are similar. Taken together, these results strongly support the conclusion that Smad3 deficiency promotes beta cell proliferation and function in *db/db* mice, which is not a secondary event due to the failure of diabetes development in the first place, but may partly contribute to the prevention of type 2 diabetes development in these animals.

The second limitation of the study is the lack of human islet data. Throughout, the study was on animal model and validated in mouse beta cell line Min6, which may not represent the same scenario in human islets. The chromatin states data certainly provided human evidence of the importance of Smad3 in human type 2 diabetes especially in islets. However, more work needs to be performed in human islets to show the proposed mechanism applies in human systems.

In summary, we clearly demonstrated the essentialness of Smad3 in beta cells by using our unique Smad3-WT/KO *db/db* mice. We further discovered a Pax6-dependent mechanism by which Smad3 promotes beta cell proliferation and function under diabetic injury. Our findings strongly suggested that islet-specific silencing of Smad3 might represent as a novel therapeutic strategy for type 2 diabetes prevention and treatment.

## Supplementary Material

Supplementary files, figures and tables.

## Abbreviations

ANOVA: analysis of variance
AGEs: advanced glycation end-products
Ang II: angiotensin II
ChIP-Seq: Chromatin immunoprecipitation-sequencing
DNTβRII: dominant negative TGF-β receptor II
IPGTT: intraperitoneal glucose tolerance test
IPGTT: intraperitoneal insulin tolerance test
Heter: heterozygous
KO: knock-out
KRBH: Krebs-Ringer bicarbonate HEPES
TGF-β: transforming growth factor-β
WT: wild-type
