# Supplementary material for: Smad3 deficiency promotes beta cell proliferation and function in db/db mice via restoring Pax6 expression
Source: Theranostics. 2021 Jan 1;11(6):2845–59. doi: 10.7150/thno.51857 (PMC7806493; doi:10.7150/thno.51857)
Supplement: Supplementary file 1 — Supplementary files, figures and tables. [file thnov11p2845s1.zip › Supplementary files/(Revision) Suppl figures and tables.pdf]

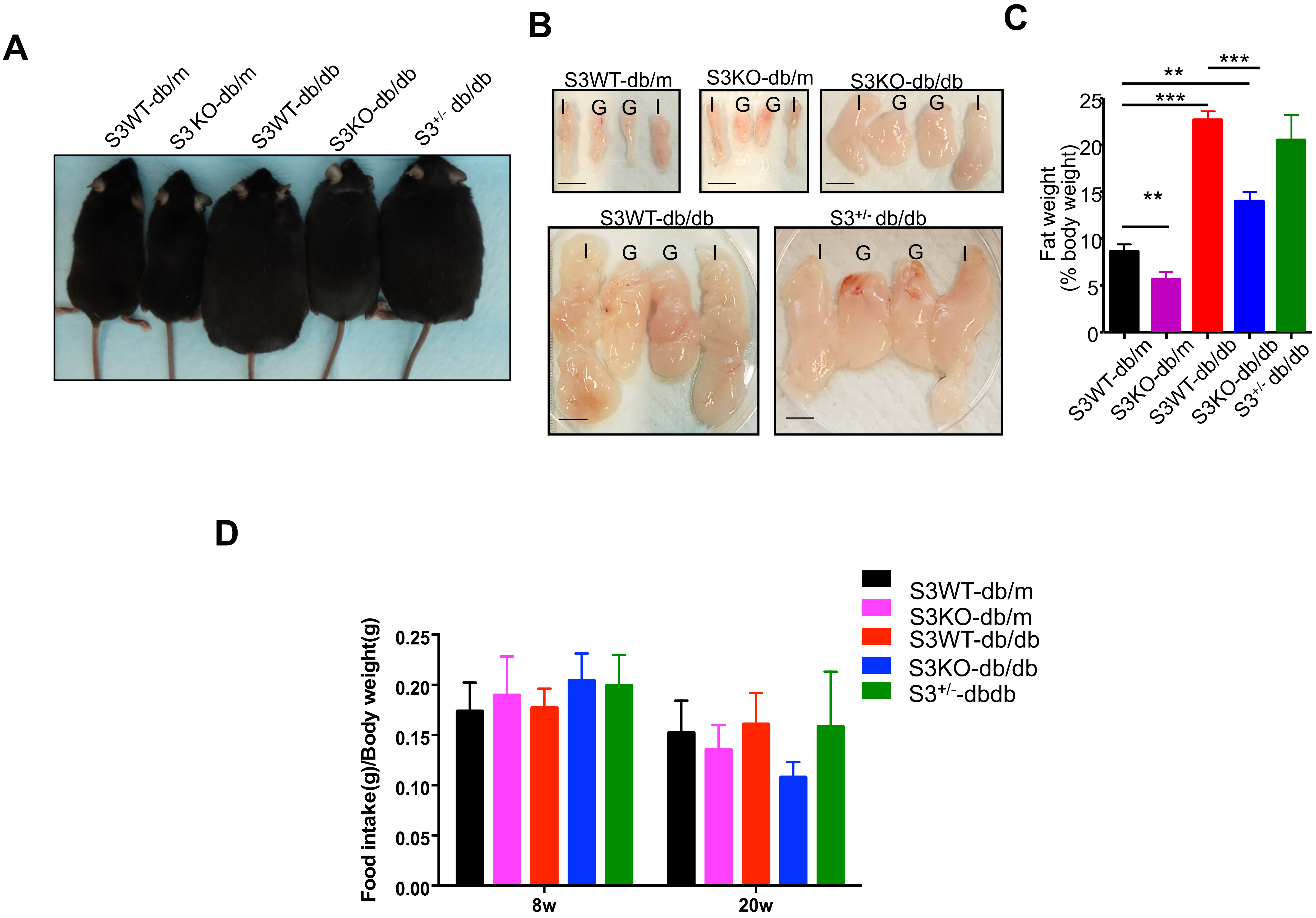

Supplementary Figure 1. Smad3 deficiency prevents the development of obesity in db/db mice. (A) Representative photograph showing mice appearance at 20 weeks of age. (B) Representative photographs showing inguinal (I) and gonadal (G) white adipose tissue (WAT) of mice at 20 weeks of age. (C) The percentage of inguinal and gonadal WAT weight of overall body weight. (D) The average food intake amount per day was normalized to mouse body weight. Data are presented as mean ± s.e.m. from groups of 8 mice. ***p*<0.01, ****p*<0.001 compared between groups as indicated, two-way ANOVA following Newman-Keuls multiple comparisons.

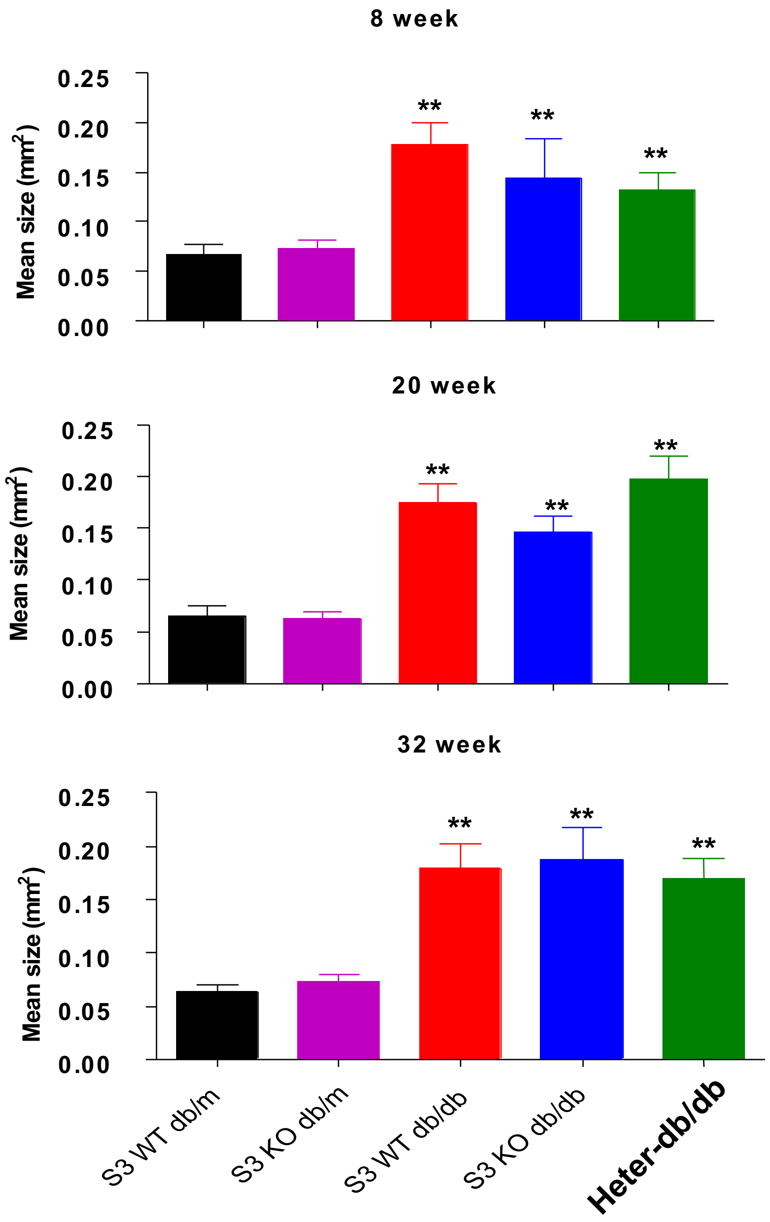

Supplementary Figure 2. Quantitative analysis of islet sizes in db/m and db/db mice. The islet sizes were significantly enlarged in the db/db mice compared to the nondiabetic db/m controls, where no significant changes were detected among db/db mice with indicated Smad3 genotypes (n=6 per group). Five non-consecutive sections of each pancreas by Image J. Data are mean ± S.E.M. **$p<0.005$ vs S3WT-db/m, two-way ANOVA following Newman-Keuls multiple comparisons.

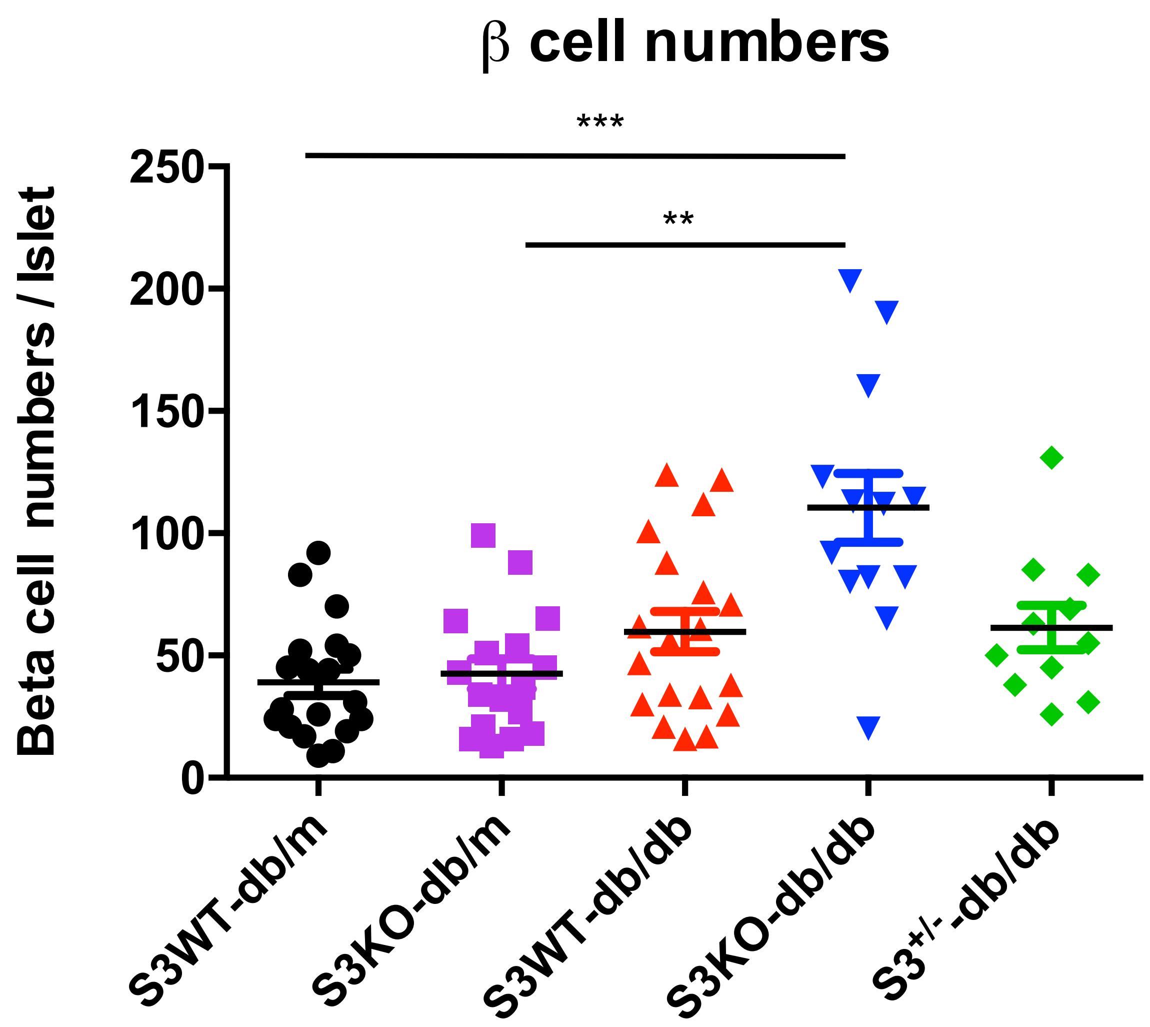

Supplementary Figure 3. Quantitative analysis of islet beta cell numbers in mice.
The average beta cell numbers per islet were significantly increased in Smad3 KO-db/db mice compared to the nondiabetic db/m controls and diabetic db/db controls (n>3 per group). Three non-consecutive sections of each mouse were analyzed. Data are mean ± S.E.M. Each dot represents one islet. **p<0.005 , ***p<0.001, two-way ANOVA following Newman-Keuls multiple comparisons.

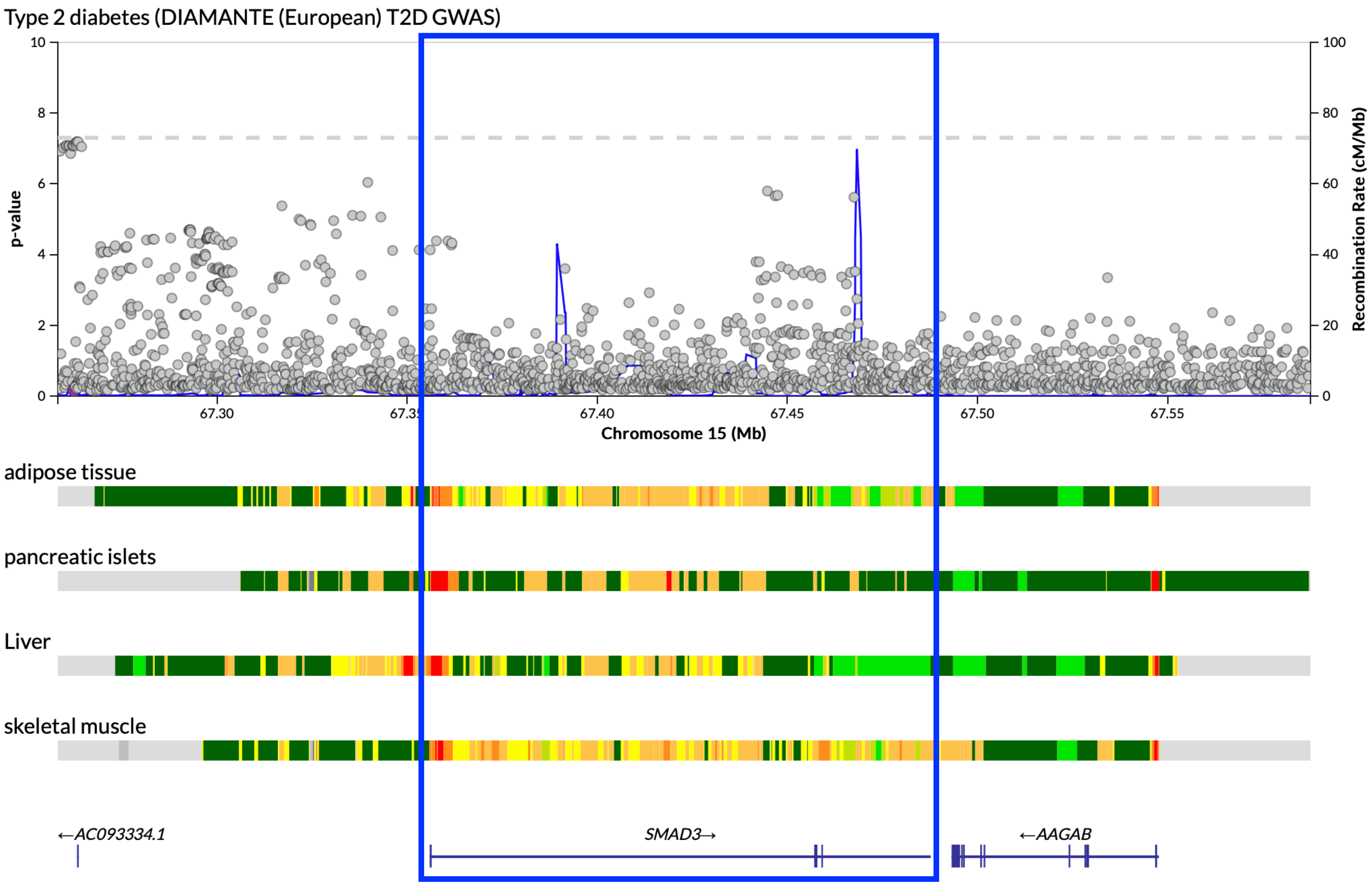

Supplementary Figure 4. Chromatin states of SMAD3 from T2D knowledge.
Upper panel: **DIAMANTE (European) T2D GWAS ;** Lower panel: The active transcription start site (Red) was especially inncreased in the Smad3 genome sequence (Blue square highlighted) in the diabetic pancreatic islets rather than adipose tissue, liver, and skeletal muscle.

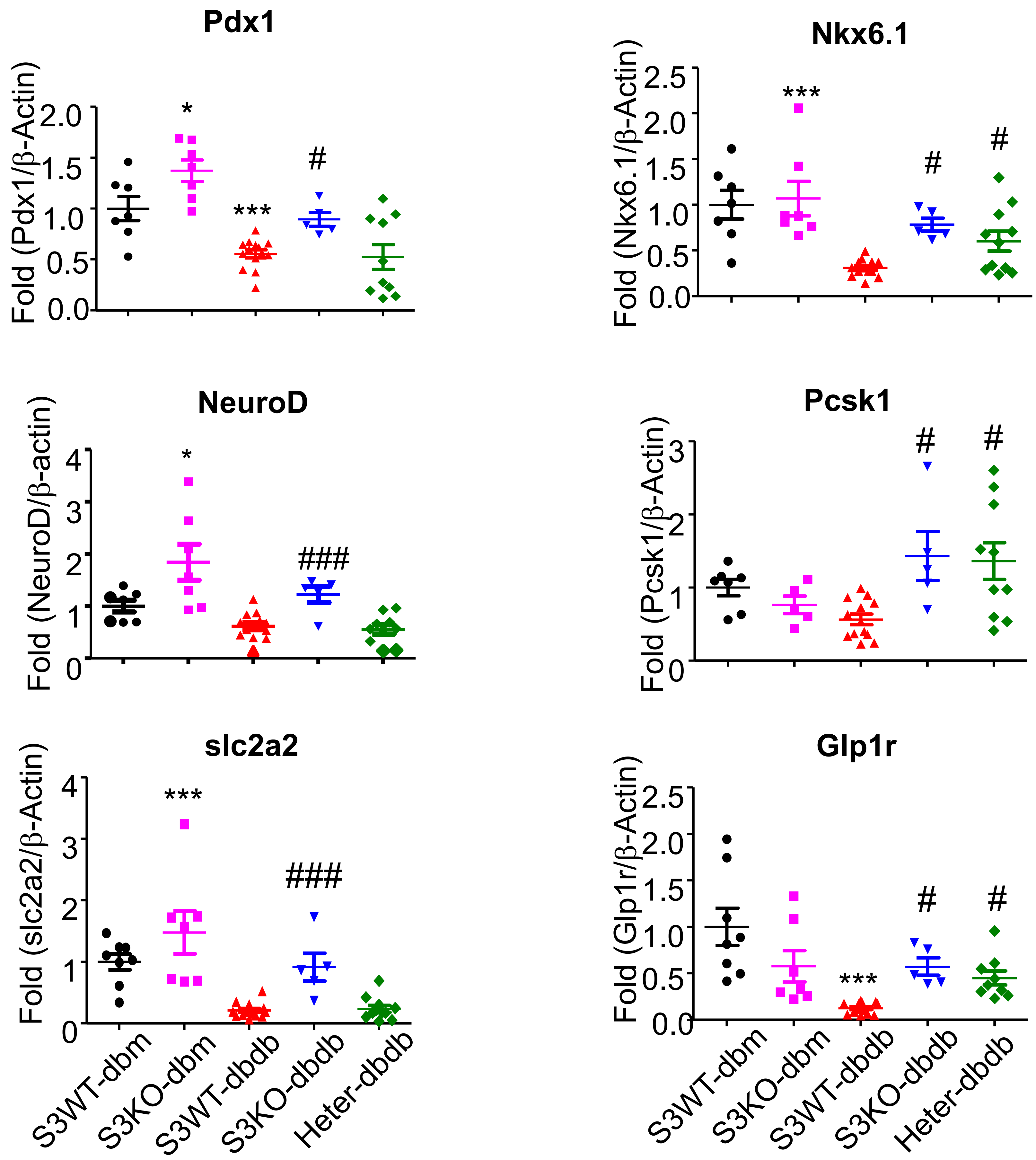

Supplementary Figure 5. The mRNA expression levels of islet development genes in mice.
Real-time PCR analysis showing the mRNA levels of individual genes related islet differentiation and function in the isolated islets from db/m and db/db mice (n>5 per group). Data are mean ± S.E.M. *p<0.05, **p<0.005, ***p<0.001 vs S3WT-db/m; #p<0.05, ###p<0.001 vs S3WT-db/db; two-way ANOVA following Newman-Keuls multiple comparisons.

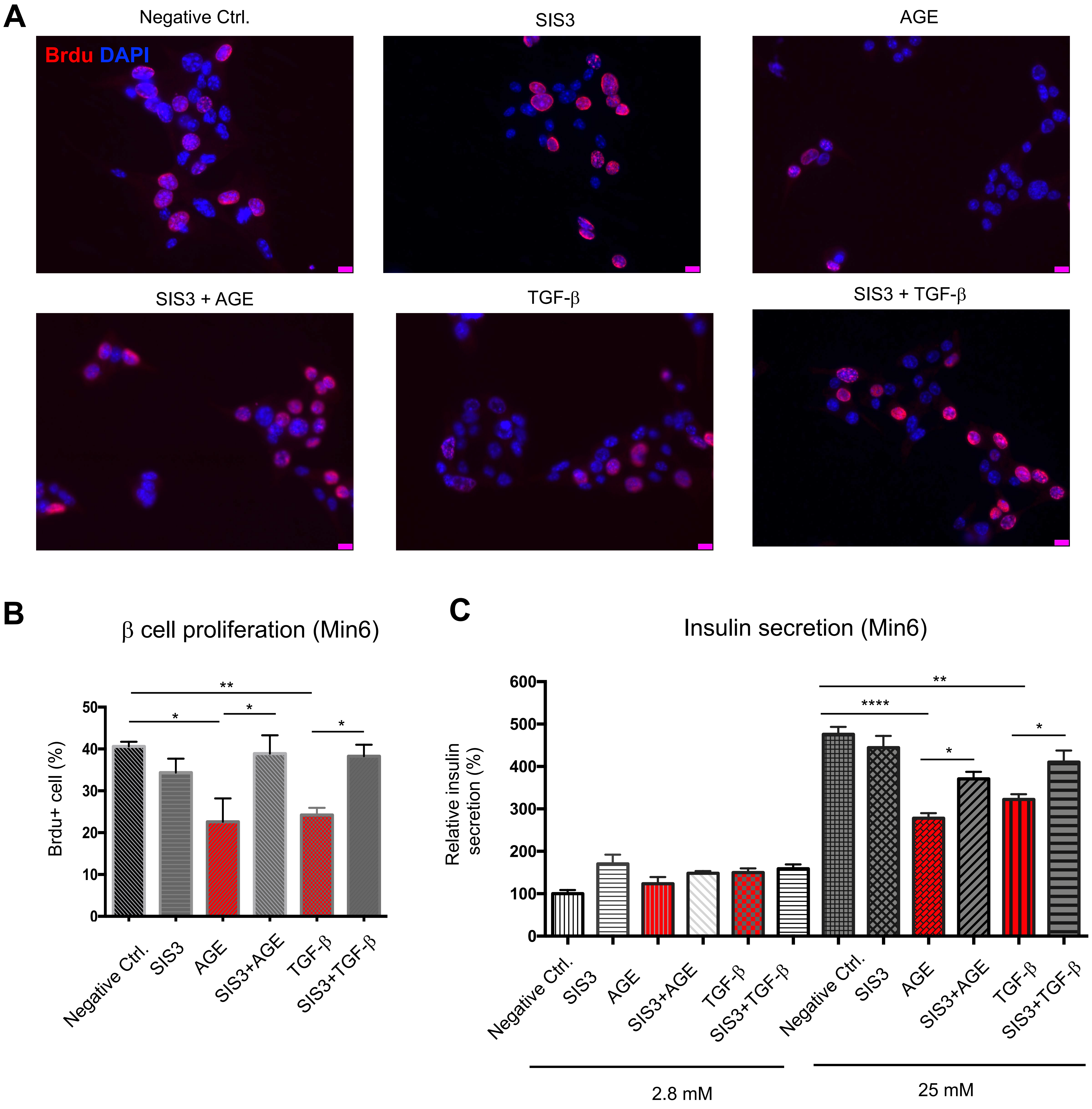

Supplementary Figure 6. AGE and TGF-β impaired cell proliferation and insulin secretion in Min6 cells.

Min 6 cells were pre-treated with or without SIS3 and stimulated with AGE or TGF-**β. (A–B)** Cell proliferation was detected by Brdu incorporation. Scale bar, 50 µm. (C) Glucose stimulated insulin secretion was detected by ELISA. Data are mean ± S.E.M. **p*<0.05, ***p*<0.01, *****p*<0.0001. One-way ANOVA following multiple comparisons.

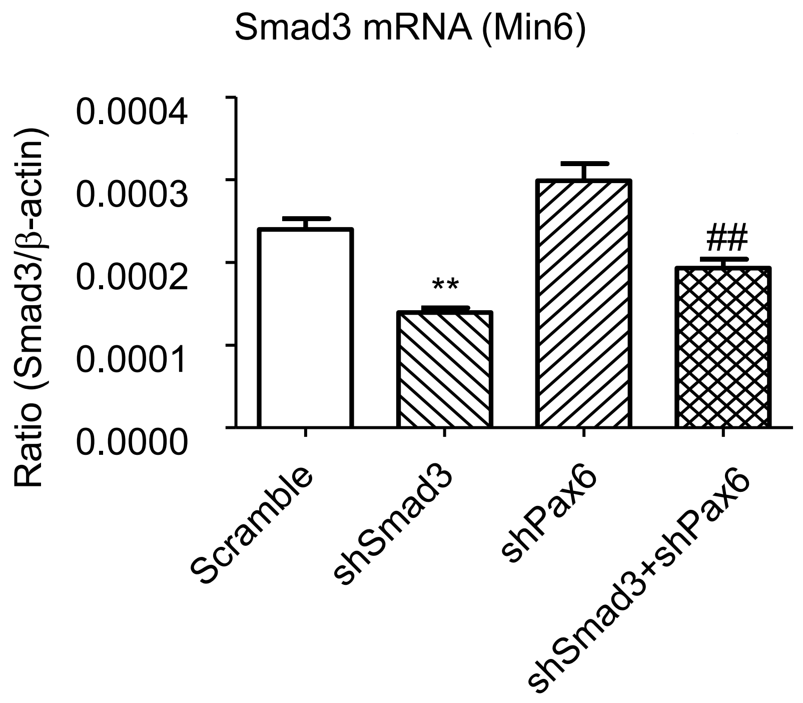

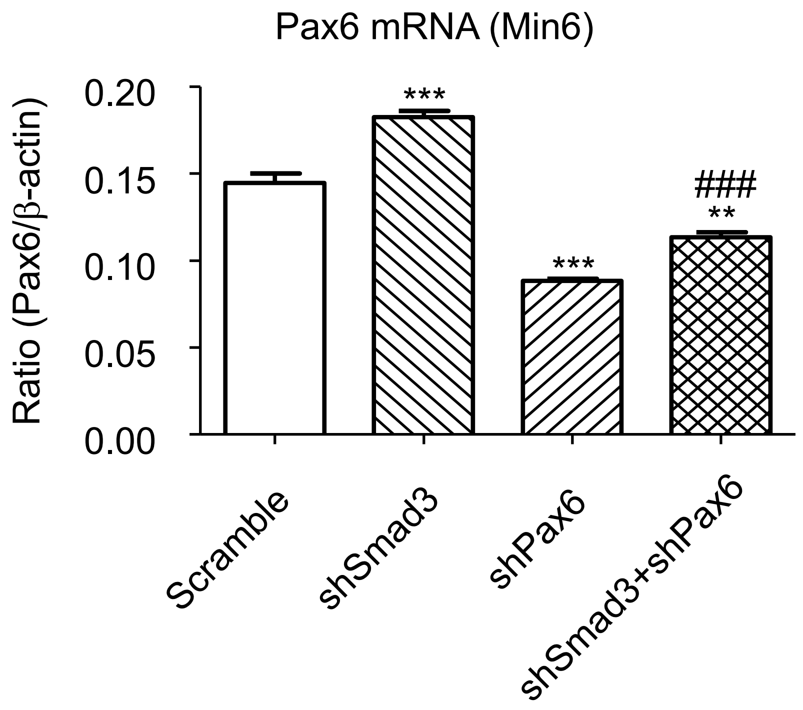

Supplementary Figure 7. Knockdown of Smad3 increased Pax6 expression on Min6 cells *in vitro*. Real-time PCR detected that Smad3 and Pax6 were successfully knockdown in the Min6 cells, where Smad3 knockdown significantly increased the Pax6 expression (n=3). Data are mean ± s.d. $**p<0.005$, $***p<0.001$ vs scramble; ##p<0.005, ###p<0.001 vs shSmad3; two-way ANOVA following Newman-Keuls multiple comparisons.

Supplementary Table 1. Genes directly and indirectly regulated by Pax6

Gene	Function	Type of regulation
Pdx1	Pancreas development<br>β cell differentiation and function<br>Insulin secretion	Direct binding (Samaras et al., 2002)
MafA	β cell differentiation and function<br>Insulin secretion	Direct binding (Raum et al., 2010)
Nkx6.1	β cell differentiation and function<br>Insulin secretion	Direct binding (Gosmain et al., 2012)
NeuroD	β cell function<br>Insulin transactivation	Direct binding (Swisa et al., 2017)
Scl2a2	β cell function<br>Glucose transport and metabolism	Direct binding (Swisa et al., 2017)
G6pc2	β cell function<br>Glucose sensing	Indirect regulation (Mitchell et al., 2017; Swisa et al., 2017)
Ins1	Insulin synthesis	Direct binding (Swisa et al., 2017)
Ins2	Insulin synthesis	Direct binding (Swisa et al., 2017)
Pcsk1	Proinsulin processing	Direct binding (Gosmain et al., 2012)
Pcsk2	Proinsulin processing	Indirect regulation (Gosmain et al., 2010)
Gipr	Glucose-stimulated insulin secretion	Direct binding (Gosmain et al., 2012)
Glp1r	Glucose-stimulated insulin secretion	Direct binding (Gosmain et al., 2012)

References

Gosmain, Y., Katz, L.S., Masson, M.H., Cheyssac, C., Poisson, C., and Philippe, J. (2012). Pax6 is crucial for beta-cell function, insulin biosynthesis, and glucose-induced insulin secretion. Mol Endocrinol *26*, 696-709.

Gosmain, Y., Marthinet, E., Cheyssac, C., Guerardel, A., Mamin, A., Katz, L.S., Bouzakri, K., and Philippe, J. (2010). Pax6 controls the expression of critical genes involved in pancreatic {alpha} cell differentiation and function. J Biol Chem *285*, 33381-33393.

Mitchell, R.K., Nguyen-Tu, M.S., Chabosseau, P., Callingham, R.M., Pullen, T.J., Cheung, R., Leclerc, I., Hodson, D.J., and Rutter, G.A. (2017). The transcription factor Pax6 is required for pancreatic beta cell identity, glucose-regulated ATP synthesis, and Ca(2+) dynamics in adult mice. J Biol Chem *292*, 8892-8906.

Raum, J.C., Hunter, C.S., Artner, I., Henderson, E., Guo, M., Elghazi, L., Sosa-Pineda, B., Ogihara, T., Mirmira, R.G., Sussel, L., et al. (2010). Islet beta-cell-specific MafA transcription requires the 5'-flanking conserved region 3 control domain. Mol Cell Biol *30*, 4234-4244.

Samaras, S.E., Cissell, M.A., Gerrish, K., Wright, C.V., Gannon, M., and Stein, R. (2002). Conserved sequences in a tissue-specific regulatory region of the pdx-1 gene mediate transcription in Pancreatic beta cells: role for hepatocyte nuclear factor 3 beta and Pax6. Mol Cell Biol *22*, 4702-4713.

Swisa, A., Avrahami, D., Eden, N., Zhang, J., Feleke, E., Dahan, T., Cohen-Tayar, Y., Stolovich-Rain, M., Kaestner, K.H., Glaser, B., et al. (2017). PAX6 maintains beta cell identity by repressing genes of alternative islet cell types. J Clin Invest *127*, 230-243.

Supplementary Table 2. Real-time PCR primers

Gene	Forward	Reverse
β-Actin	AGAGGGAAATCGTGCGTGAC	CAATAGTGATGACCTGGCCGT
Pax6	CCCATGCAGATGCAAAAGTC	GCCAGTCTCGTAATACCTGC
Pdx1	TTCAACATCACTGCCAGCTC	GAAATCCACCAAAGCTCACG
Nkx6.1	TCAGGTCAAGGTCTGGTTCC	CGATTTGTGCTTTTTCAGCA
NeruoD	CAAAGCCACGGATCAATCTT	CCCGGGAATAGTGAAACTGA
Slc2a2	CCACATACATCAGGAATCTTGC	TGAGACGGTAGACCAGGAAAG
Ins2	GAGCAGGTGACCTTCAGACC	TTCATTGCAGAGGGGTAGGC
Pcsk1	GCTCCATCTTTGTCTGGGCT	TCGCTGGTCTGTGTAATCACC
Glp1r	TTTGTGATGGACGAACACGC	CACTTGAGGGGCTTCATGCT
